# The Impact of the Direct Participation of Workers on the Rates of Absenteeism in the Spanish Labor Environment

**DOI:** 10.3390/ijerph17072477

**Published:** 2020-04-05

**Authors:** Raúl Payá Castiblanque

**Affiliations:** Department of Sociology and Social Anthropology. University of Valencia, Av. dels Tarongers, 4b, 46022 Valencia, Spain; raul.paya@uv.es

**Keywords:** work absenteeism, direct participation, prevention management, multinomial logistic regression, preventive culture, co-management

## Abstract

The aim of this research was to study the relationship between the different levels of direct participation of workers (passive, consultative or active-delegated) in risk prevention management with the levels of absenteeism in Spain. To this end, a transversal study was carried out using microdata from the Second European Survey of Companies on New and Emerging Risks (ESENER-2-Spain, 2014) with a master population of 3162 work centres. A multinomial logistic regression model was carried out, with the dependent variable being the levels of absenteeism and the independent variables, the participation indicators and preventive management, calculating the adjusted odds ratio (aOR) between all the independent and control variables, with their corresponding 95% confidence intervals (95% IC). The results obtained showed how the active-delegative participation of workers in the design and adoption of psychosocial risk prevention measures reported 2.33 less probabilities of having a very high or fairly high level of absenteeism (aOR = 0.43; 95%IC:0.27–0.69). However, having documented aspects of preventive management (plan, risk assessment, planning measures) did not have any impact on absenteeism levels, which shows that we can fall into an unrealistic institutional mirage of security with active policies of co-education or co-management being necessary to reduce absenteeism.

## 1. Introduction

The transformations produced in the world of work derived from labor deregulation policies or the use of robotics and artificial intelligence in production processes are responsible for the emergence of new labor risks of psychosocial origin [1,2,3]. Psychosocial risks, according to the International Labour Organization (ILO) [4], are effects derived, on the one hand, from the dynamic interaction between human relationships (communication systems, social support, etc.) and, on the other hand, from the organization of work (production rates, work schedules, job design etc.). Such risks of psychosocial origin have an increasing incidence within all occupational risks in Europe in general, and Spain in particular. This sense, the latest report of the Spanish Strategy for Occupational Health and Safety 2015–2020 [5] compares the evolution of the different occupational risk factors with the data obtained from the National Surveys of Working Conditions of 2007 and 2011. This report shows an increase in workers’ concern about job instability (from 21.9% in 2007 to 51.4% in 2011), the intensification of quantitative requirements (having a lot of work happens from 20.3% to 24%; working with demanding deadlines goes from 33.5% to 35%; working very fast goes from 44% to 46%), sensory (keeping the level of attention high or very high passes from 67% to 77.6%), cognitive (attending several tasks at the same time goes from 41.2% to 45.3%) and emotional (dealing directly with the public goes from 58.6% to 64%).

There is ample scientific evidence of the relationship between the presence of psychosocial risks in organizations and the appearance of negative effects on workers’ health, both psychosomatic (anxiety, depression, stress, sleep problems, etc.) [6,7] and cardiovascular [8,9]. The World Health Organization (WHO) has estimated that, as a result of increased exposure to psychosocial risk factors, anxiety and depression will be the main cause of absenteeism in Spain from 2020 [10]. However, several Spanish legal studies have shown that psychopathic pathologies are systematically excluded from official records of occupational accidents and diseases [11,12,13]. In fact, it is estimated that “if the approximately 400,000 annual cases of accidents due to common contingencies of psychological or psychiatric origin were added to the recognized occupational diseases, the total figure would increase by almost 40%” [12]. Therefore, this research focuses on the study of the determinants of absenteeism and does not take as a reference the official records of occupational accidents. Workplace absenteeism is a broader construct than that of accidents at work, since it includes attitudinal, economic, organizational, work environment and job satisfaction factors [14] that allow for the interrelationship of individual and organizational motivation variables, being much more appropriate for measuring the impact of psychosocial risk factors on workers’ health [15].

From the point of view of the determinants of health in the prevention of occupational risks, many studies have shown that the indirect participation of workers through trade union representatives or specialised occupational health representatives (prevention delegates or health and safety committees) improves the rates of management of occupational risk prevention [16,17,18] and therefore has a positive effect on reducing the incidence rates of occupational accidents [19,20,21]. There are even comparative studies that have found an inverse relationship between union strength and occupational accident levels among European countries [22,23,24]. More recent research would go some way to criticizing approaches that focus solely on studying the direct relationship between the presence of collective representation in companies and risk prevention document management or its impact on reducing incidence rates for accidents at work [25,26,27,28]. These studies from a holistic perspective seek to understand both the external and internal determinants of the workplace that influence the effectiveness of worker participation. In this sense, the following external determinants can be identified: macrocontextual factors, related to policies and systems of legal regulation of occupational health [28,29], such as the capacity of representatives and workers to paralyze productive activity in the face of serious or imminent risk [30]; the promotion of regulations that reward the integration of the prevention management system through its own means to facilitate participation [31]; the promotion of participation through the requirements of the labour inspection [32], regulations that do not systematically make technicians responsible for the prevention of accidents in companies to prevent expert knowledge from blocking participation [33]; or the promotion of policies for the representation of interests that facilitate decentralised self-regulation through the participation of autonomous trade unions [27,34]. The internal factors that influence the effectiveness of participation are business leadership and willingness to promote a participatory culture [35,36,37,38,39,40]; training and empowerment of workers and their representatives to collectively challenge unsafe situations [37,38,39]; the size of the workplace and the sector of activity to the extent that participation is greater in larger workplaces and in industries where occupational risks are more evident [18,27,41]; in addition, greater capacity for participation will exist when specialized occupational health representation is unionized [42] or has strong external support from the union [43,44].

Despite constituting a consolidated line of research, the above-mentioned studies have focused, on the one hand, on the management of the traditional risks of industrial safety and hygiene in the workplace responsible for the generation of occupational accidents and, on the other hand, their main study objective has been to demonstrate that the indirect and representative participation of workers is one of the main determinants of the reduction of occupational accidents and the improvement of working conditions. However, less studied is the impact of direct worker participation in the management of psychosocial risks on absenteeism levels. In fact, Walters and Wadsworth [27] state in a recent study that “here, it needs to be acknowledged that while there is a body of reasonably robust evidence demonstrating the effectiveness of the operation of statutory approaches to worker representation and consultation on safety and health and what makes it so, no comparable body of evidence exists on the role of direct participation.” In this sense, the direct participation of workers has been studied together with other dimensions of work management, other than risk prevention, finding controversial results. On the one hand, direct participation improves the quality of the psychosocial environment and working life [45,46,47,48,49,50], but, on the other hand, participation has also been associated with self-exploration and labour intensification, to the extent that decentralization of decision-making capacity produces a process of indoctrination among workers themselves and disproportionate expectations from management regarding the commitment of their employees [51,52,53,54,55].

In relation to the above, worker participation from the point of view of industrial democracy must be understood as a process of decentralization of decision-making in risk prevention management [56] and, therefore, has a political component that cannot be separated from problems of authority and legitimacy [57]. Thus, depending on the levels of decentralization of decision-making, a distinction can be made between (a) passive participation occurs when workers are passive recipients of information and training on the occupational risks to which they are exposed; (b) intermediate or consultative participation occurs when management talks and discusses with workers aspects of preventive management but the final decision on action measures is taken by management; (c) active or delegated participation occurs when the management delegates to the workers the decision making and design of the preventive action measures talking on this occasion about co-management or co-determination processes [57,58,59,60].

For the purposes of this research, the management of occupational risk prevention must be understood as a legal process established in Law 31/1995 on the Prevention of Occupational Risks (LPRL) [61] in compliance with Framework Directive 89/391-CEE, which determines that companies are obliged to manage prevention along three lines (art. 16): (a) design and implementation of prevention plans to establish the resources, responsibilities and preventive procedures; (b) carrying out the corresponding evaluations; and (c) planning preventive action measures to eliminate or reduce the risks observed in the evaluation process. As regards the phase or period of time in which worker participation in preventive management takes place, two periods can be identified. On the one hand, the two initial phases referring to participation in the elaboration of the prevention or risk identification plan in the evaluation and, on the other hand, the final phase of the process, referring to participation in the design of the preventive action measures [62]. Studies related to traditional risks have shown that “deeper” levels of participation (active and at the end of the preventive management cycle) have a greater quantitative impact on the reduction of occupational accidents, in relation to “passive” participation based on the transmission of information at the beginning of the preventive management system [21]. However, given the power problems in the decentralization of decision-making described above, in the end “there is a lot of information and consultation and little or no real participation” [63]. In the same way that the LPRL establishes the company’s obligation to integrate the prevention of occupational risks in the workplace, it also determines the right to employee participation in terms of information (Article 18 of the LPRL), consultation (Article 33 and Chapter V of the LPRL) and active employee participation (Article 39.1a and Chapter V of the LPRL), even extending the protection provided by the Framework Directive [64]. However, in Spain, the combined effect of an international context dominated by a liberal economic and political paradigm [27] with a fragmented productive fabric in small companies in which management adopts paternalistic and authoritarian behaviour [65,66,67] undermines any real participation in matters of occupational health and safety, often becoming a bureaucratic process to formally comply with the law [41,42,43,44,45,46,47,48,49,50,51,52,53,54,55,56,57,58,59,60,61,62,63,64,65,66,67,68]. It has been mentioned that in the determinants of participation effectiveness the presence of union representatives drives real participation [43,44]. For this reason, several studies have shown that supra-business systems of representation through territorial prevention delegates have been developed so that the union can penetrate the smallest companies, obtaining positive effects [69,70,71]. However, in Spain there are few systems of representation of this type [66,69,70,71].

**Figure 1:**
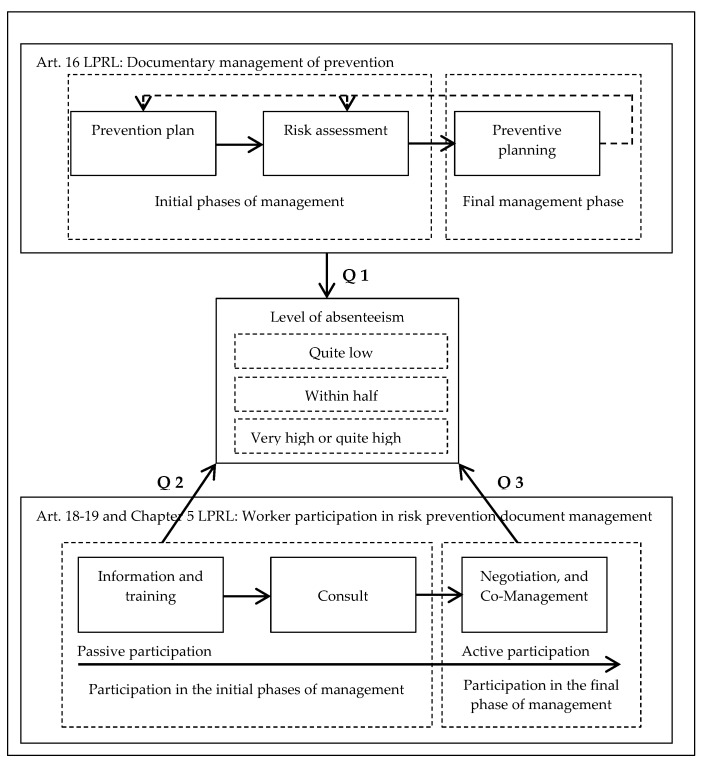
Participatory management system for the prevention of occupational risks. Q1: first research question; Q2: second research question; Q3: third research question; Art. 16: article 16 of the law of 31/1995 on the prevention of occupational hazards (LPRL); Art. 18-19 and Chapter V: articles 18, 19 and Chapter V of the law of 31/1995 on the prevention of occupational hazards (LPRL).

In relation to the above, this study starts from the hypothesis that the existence of a regulatory framework that obliges organisations to have formal documentation that accredits compliance with the law in risk prevention management does not guarantee the effectiveness of the system, while those organisations that promote a participatory preventive culture in the management of psychosocial risks have a positive impact on absenteeism levels. This hypothesis is in line with the postulates of the historical American movement known as Safety First [72,73], which defended the idea that the reduction of occupational accidents derived from traditional risks should be achieved by activating a culture of participatory prevention within the organization. However, Western European countries have historically defended an environmental hypothesis based on the principle of protection of the worker by the public authority through national regulations to reduce occupational accidents [72,73]. In view of this hypothesis, we propose to answer the following questions about the process of participatory management of occupational risk prevention (Figure 1): first question Q1) is it sufficient to have documented prevention management (plans, risk assessments and prevention planning) or on the contrary, does the mere management not guarantee the effectiveness (absenteeism) of the system, being necessary the participation of the workers in the process?. Second question Q2: Will the passive participation (information and consultation) of workers in the management system be sufficient to reduce absenteeism, or, third question Q3: Will active participation of workers be required in the design and adoption of prevention measures to guarantee the reduction of absenteeism?

Taking into account the background, in order to investigate the hypothesis and answer the questions raised, this research studies the relations between (1) the levels of direct participation (passive, consultative and delegated) of the workers in preventive management and (2) the indicators of the management process itself (plans, evaluations and planning) with (3) the levels of absenteeism in the Spanish working environment. In short, the aim of this research is to demonstrate that bureaucratic management of prevention alone does not reduce absenteeism and, however, when the subjectivity of the workers is taken into account by allowing their direct participation in the process of prevention management, that is when absenteeism rates are really reduced.

## 2. Materials and Methods

### 2.1. Source and Sample

To develop the objective of this research, a transversal study has been carried out through the statistical analysis of the microdata from the Second European Survey of Companies on New and Emerging Risks (ESENER-2-Spain, 2014) [74] prepared by the National Institute of Safety and Hygiene in the Workplace (INSHT). The survey has a population of 3162 work centres in Spain with five or more workers from all activity sectors, except for sections T (Domestic activities) and U (Extraterritorial organisations) of the National Classification of Economic Activities (CNAE Rev.2). The survey has been considered to be representative of the entire business fabric of the national territory for two reasons. Firstly, the ESENER-2-Spain survey, as observed, includes practically all sections of the CNAE, and secondly, it presents a confidence level of 95.5% (two sigmas) and a sampling error of ±1.77%, and therefore presents adequate confidence levels to validate the statistical results found in this study. On the other hand, it should be added that the fieldwork for the preparation of the survey was carried out between 14 July 2014 and 20 October 2014, with the interviewee being “the person who knows most about occupational safety and health” in the opinion of the company manager [74], which in turn generates better levels of confidence because the main source of obtaining the data is people who specialize in issues related to occupational safety and health management. To conclude, it should be mentioned that the ESENER-2 survey has been selected over other data sources such as the National Surveys on Working Conditions (ENCT) because the ESENER-2 questionnaire includes questions related to both the different phases of preventive management (prevention plan, risk assessment and planning of preventive actions) and the direct participation of workers in this management (passive participation, consultation and active-prospective participation), as well as information on the levels of absenteeism in the work centres.

### 2.2. Dependent Variable

With regard to the dependent variable used to measure the level of absenteeism, the following question was selected from the questionnaire: Q450.- “How would you describe the level of absenteeism at your workplace compared to other workplaces in the industry? The possible answer alternatives were: “Is it very high, quite high, within the average, quite low or very low”. As can be seen, the question had five answer alternatives, but, however, in order to carry out the present study, the dependent variable was recoded and transformed into four answer alternatives (1 = very high or quite high; 2 = within the measure; 3 = quite low; 4 = very low). Specifically, the responses corresponding to the very high and fairly high levels were considered within the same category, since 151 cases were recorded between both response alternatives (52 with very high absenteeism and 99 fairly high), representing 4.8% of the total and, therefore, preventing a broader stratified statistical analysis to measure the level of absenteeism.

### 2.3. Independent Variables and Adjustment Covariates

Seventeen dummy variables were selected from the database, corresponding both to the levels of direct worker participation (6 indicators) and to the different phases of preventive management (11 indicators). The indicators used to measure the levels of direct participation were: (a) passive participation was measured, on the one hand, through question Q256_5 which asked whether the workers had received the information corresponding to the results of the risk assessment and, on the other hand, whether they had received training on psychosocial risks (Q166_3); (b) the consultation or intermediate level of participation was measured through question Q358 asking about the presence or absence of discussions on preventive issues in staff or team meetings and, question Q350 asking whether the management of the company talked to the workers and their representatives about preventive aspects; and (c) active and delegated participation was measured by two indicators. The first, of a general nature, was measured through question Q258b “If measures need to be taken following a risk assessment, do workers normally participate in their design and implementation”, and the second, specific to psychosocial risk management, was measured through question Q305 “Did workers participate in the design and adoption of measures to prevent psychosocial risks?”.

The 11 variables corresponding to the preventive management rules were selected according to the three phases of the management system, in order to find the presence or absence in the workplaces of prevention plans (four indicators), risk assessments (three) or preventive action planning measures (four). With reference to the indicators corresponding to the prevention plans, both general variables were used (Q156: there is a specific annual budget for prevention measures and equipment) and specific variables on psychosocial risks (Q300: there is an action plan to prevent work-related stress; Q301: there is a procedure to deal with cases of harassment or mobbing; and Q302: there is a procedure to deal with threats, insults or aggression by persons outside the organisation). As for the second phase of the preventive management system corresponding to carrying out risk assessments, question Q250 was used as an indicator that brings together all the occupational risk factors (safety, hygiene, ergonomics and psychosociology). Furthermore, the analysis included the specific indicators corresponding to the assessment of psychosocial risks both in their relational dimension (Q252_5: Relations between the worker and his/her supervisor) and in the organizational aspects such as working hours, breaks or shifts (Q252_6). To measure the last phase of preventive management corresponding to the planning of actions to eliminate or reduce exposure to the risk factors identified in the assessment phase, question Q303 was used, asking about prevention measures carried out to prevent psychosocial risks in the last 3 years. In particular, the question referred to the presence or absence of measures such as reorganization of work to reduce demands and work pressure (Q303_1), confidential counselling for workers (Q303_2), application of conflict resolution procedures (Q303_3) and intervention in case of long or irregular working hours (Q303_4).

To measure the specific effect of the levels of direct worker participation on the levels of absenteeism, a set of control variables have been used based on the conclusions identified in previous studies in order to avoid interference bias from other variables or to avoid spurious relationships. In this sense, as identified in the theoretical framework, in the previous literature inverse relationships have been found between the levels of occupational accidents and the indirect or representative participation of workers, both general (unitary and union) and specialized in occupational health (prevention delegates and health and safety committee) [19,20,21], so question Q166 corresponding to the presence or absence of the different systems of representation in the workplace was included as an adjustment variable. The following questions were also included as an adjustment variable: (a) question Q165 that asked whether or not the work centre had received a visit from the labour inspection in the last three years; (b) question Q451 referring to the economic situation of the company (very good; quite good; neither good nor bad; and, very bad); and (c) question Q104 corresponding to the size of the work centre (from five to nine workers; from 10 to 49; from 50 to 249; and, from 250 or more workers). These variables were included in the statistical model derived from previous studies that have identified that in smaller companies, with economic difficulties and that do not receive visits from the labour inspectorate, the levels of management of psychosocial risks are less intense [74,75].

Finally, it should be mentioned that the sector of activity of the workplace was also included as an adjustment covariate (Q112) insofar as the sectorization of occupational risks may affect both levels of absenteeism and levels of participation and management of psychosocial risks [76]. The following shows the relationship of the dependent, independent and adjustment variables included in the statistical analysis (Table 1).

### 2.4. Statistical Analysis

The technique used to measure the effect of participation and preventive management indicators on absenteeism levels was multinomial logistic regression, derived from the ordinal character of the dependent variable. This technique was used to classify the subjects according to the values of a set of predictor or control variables, that is, for the purposes of this research, the multinomial logistic regression made it possible to identify which participation and preventive management variables had the greatest incidence and impact on each stratum of absenteeism. Specifically, the associations between each level of work absenteeism with each of the independent indicators of participation and management were estimated by calculating the odds ratio (aOR) adjusted for the rest of the independent and control variables, with their corresponding 95% Confidence Intervals (95% CI). For the dependent variable, the reference category was the very low level of absenteeism. For the independent variables, the reference category was absence of participation or absence of preventive management. All calculations were performed with SPSS version 26 statistical software (IBM Corp, Armonk, NY, USA).

## 3. Results

In general terms, the results obtained (Table 2), on the one hand, showed that the indicators corresponding to the management of occupational risk prevention, both general and specialized in psychosocial risks, were not significantly related to the different levels of absenteeism, but, on the other hand, it was found that the participation of workers in these preventive management processes did have a positive impact on reducing absenteeism.

Specifically, with regard to preventive management indicators, when comparing organisations with very low levels of absenteeism with those with very high or fairly high levels of absenteeism, it was observed that no variables related to prevention plans, risk assessment and the planning of preventive action measures were statistically significant. However, in the intermediate levels of absenteeism (quite low/within the average) some indicators of preventive action such as the availability in the workplaces of procedures to deal with cases of harassment or bullying (aOR = 1.69; 95%IC95:1.14–2.49), the implementation of conflict resolution actions (aOR = 1.40; 95%IC:1.07–1.83) or confidential advice to workers (aOR = 1.41; 95%IC:1.10–1.81 for the fairly low level; aOR = 1.30; 95%IC: 1.01–1.68 for the mean) were associated with higher rates of absenteeism compared to organizations with a very low level of absenteeism.

By focusing on participation indicators, relationships of varying intensity were found according to different levels of participation and absenteeism. In reference to passive participation, it was identified as the organizations that inform workers about the risks of their jobs were 1.41 less likely to have a fairly low level of absenteeism (aOR = 0.71; IC95%:0.57–0.87) or about 1.56 less likely to refer to a level of absenteeism within the sector average (aOR = 0.64; IC95%:0.51–0.79) with respect to organizations with a very low level of absenteeism. The other passive indicator of participation showed a similar pattern, in that work centres where workers were trained on psychosocial risks had a 1.32 lower probability of having low absenteeism (aOR = 0.76; IC95%:0.51–0.95. However, passive participation was not predictive of higher levels of absenteeism.

In this regard, the only indicators that showed a positive effect in reducing the highest levels of absenteeism were those of active or delegated participation. Thus, the organizations that involved workers in the design and application of preventive action measures after the identification of the general risks in the assessment document reported a 1.47 lower probability of having a level of absenteeism within the sector average (aOR = 0.68; 95%IC95:0.62–0.89). However, the most relevant finding was to identify as the only indicator that positively impacted on the higher levels of absenteeism the active participation of workers in psychosocial risks, since in the workplaces where they participated in the design and adoption of measures to prevent psychosocial risks they were 2.33 less likely to have a very high or quite high level of absenteeism (aOR = 0.43; 95%CI:0.27–0.69). In addition, this delegated participation specializing in psychosocial risks also had a positive impact on both the levels of absenteeism within the organization’s sectoral measure (aOR = 0.76; 95%CI:0.58–0.99) and the low level of absenteeism (aOR = 0.66; 95%CI:0.51–0.87). However, indicators related to the intermediate or consultative level of prevention showed contradictory results. In this sense, on the one hand, in reference to the indicator of the talks on preventive matters by the managers with the workers, no statistically significant relationship was found with the absenteeism levels, but, on the other hand, the talks between the workers in the staff or team meetings showed a negative impact on the absenteeism in the two lower levels (aOR = 1.24; 95%IC:1.01–1.54 at the fairly low levels; and, aOR = 1.71; 95%IC:1.36–2.14 within the mean) and at the higher levels (aOR = 1.54; 95%IC95:1.04–2.29).

**Figure 2:**
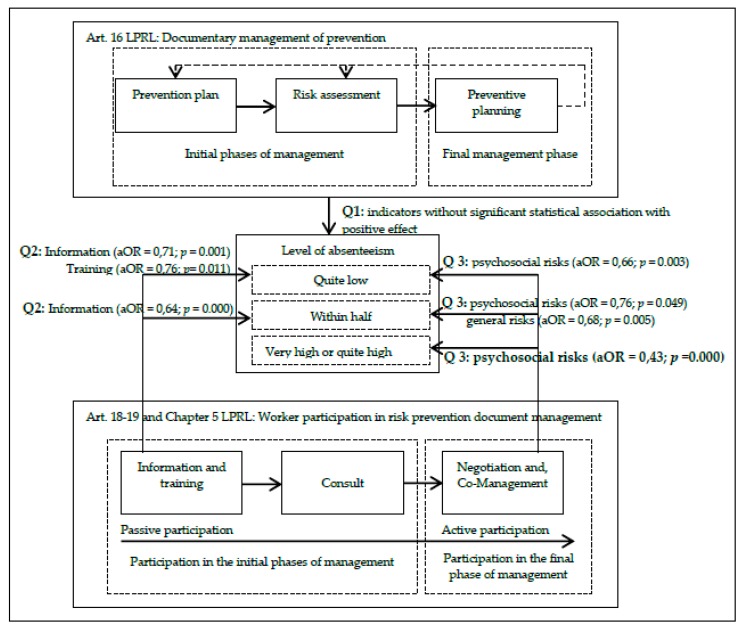
Results of the Participatory Management System for the Prevention of Occupational Risks. Q1: first research question; Q2: second research question; Q3: third research question; Art. 16: article 16 of the law of 31/1995 on the prevention of occupational hazards (LPRL); Art. 18-19 and Chapter V: articles 18, 19 and Chapter V of the law of 31/1995 on the prevention of occupational hazards (LPRL); aOR: adjusted odds ratio; *p*: *p*-value at a 95% significance level.

If we return to the flow of the process of participatory preventive management (Figure 1) and answer the questions posed with the results of the analysis in Table 2, we can answer the three initial questions of this research. Thus, by organizing the results obtained in the flow or process of participatory management (Figure 2) it can be seen that there is no positive effect on the level of absenteeism in the companies that manage the prevention of occupational risks, i.e., the presence or absence of a documented prevention plan, a risk assessment or the design of prevention measures does not differentiate the level of absenteeism among the companies (Q1).

However, the fact that workers have a passive role (information and training) in the first phases of the management process (prevention plan and risk assessment) means that companies are less likely to have intermediate levels of absenteeism (Q2), but to ensure that companies have a very low level of absenteeism and reduce the probability of them being at the highest levels in the sector, workers must be actively involved and at the end of the preventive management process in the design and implementation of psychosocial risks, but not, on any other occupational health and safety risks (Q3).

## 4. Discussion

The aim of this research was to study the relationship between the levels of direct participation of workers in the management of risk prevention systems and the indicators of preventive management itself with the levels of absenteeism. In the same way that previous studies have shown how indirect participation of workers through collective representation systems has a positive impact on the management of traditional industrial health and safety risks [16,17,18] and the reduction of the rate of occupational accidents [19,20,21,22,23,24]; the present study, in line with these findings, has found how the direct involvement of workers in managing psychosocial risks has a positive impact on reducing absenteeism rates.

The fact that direct worker participation improves absenteeism levels could be explained both by the improvement of the psychosocial environment and by the motivation generated by the very fact of participating in decision-making [45,46,47,48] as well as by the better identification of occupational risks in the evaluation and preventive planning processes, insofar as workers are the ones who best know the risks to which they are exposed and, therefore, it is necessary for the subjectivity of the working class to complement the technocratic vision of occupational health experts, especially with regard to psychosocial risk factors [58]. However, these statements need to be studied and verified in future research.

As for participation levels, the results obtained have shown similar patterns to those of previous studies [21] in that passive participation (information and training) in the first preventive management processes (plans and evaluations) has shown positive results at low and intermediate levels of absenteeism, while at higher levels it has not been predictable. However, direct and delegated active participation at the end of the preventive process (participation in the design and application of action measures) shows very positive results at the highest levels of absenteeism. Specifically, the most predictive indicator was active participation in psychosocial risks and not in any other type of occupational risk, which in turn could be explained by the association of absenteeism with the psychosocial risk factors responsible for psychosomatic pathologies [6,7].

With regard to the preventive management dimension, the results obtained have shown that they do not have a differential impact on the levels of absenteeism. This finding confirms the hypothesis of this research to the extent that having plans, risk assessments or documented preventive planning in organizations to comply with the law does not guarantee a reduction in the levels of absenteeism. Only when there is active and delegated participation in the design and implementation of the management system will the best results be achieved in terms of absenteeism. However, in Spain 88.8% of work centres have stated that they have a risk assessment document, while in only 34.5% of cases have workers participated in the design of prevention measures for psychosocial risks (Appendix A). This situation could pose a major problem for organizations in that they may fall into a kind of institutional mirage of safety when drawing up formal documents, but, nevertheless, have no real effectiveness arising from the lack of development of a participatory preventive culture within the company. This conclusion could be extended to practically all the countries of the European Union insofar as 77.2% of European workplaces have a documented risk assessment, but nevertheless the direct participation of workers in the management of psychosocial risks falls to 64.6% [74]. However, the Nordic countries (Sweden and Denmark) and some European centres such as Austria have direct participation rates in psychosocial risk management of 75.7%, 79.4% and 78.4%, respectively [74]. It is likely that in these countries, as a result of their corporate structures, tripartite social consensus and a historical culture of cooperation between capital and labour, higher levels of participation will be promoted [22]. All in all, the need to activate participative preventive cultures in Spanish organisations is highlighted, with one of the differential elements for its activation being business leadership [77,78]. In fact, the Spanish Occupational Health and Safety Strategy 2015–2020 includes among its measures to improve the preventive culture, the training and capacity building of employers, as well as other actions such as the implementation of mechanisms to improve internal communication in organisations and even to promote preventive training in the initial stages of the educational system [5].

### Limitations

The study has some limitations, so the results should be interpreted with caution. Firstly, although the survey controls for the degree of subjectivity of responses with the choice of an occupational health expert in the workplace, there may be subjective biases that direct their opinion towards what is considered socially accepted. Secondly, it is a cross-sectional study, which limits the inferences about the relationships between the variables and, therefore, prevents establishing their directionality. However, the results can be considered valid to the extent that they are controlled by variables that have previously been shown to be related to indicators of participation, management and absenteeism. Finally, the survey contains a limited number of questions related to the direct participation of workers in the preventive management process, especially with regard to intermediate or consultative participation. In this sense, the intermediate participation indicator related to management discussion with workers has not been shown to be statistically significant, while in the other indicator, the places where preventive issues were discussed in personnel or team meetings identified higher levels of absenteeism. It would be advisable to expand the number of indicators of consultative participation in future ESENER surveys. However, the fact that there is greater absenteeism in organisations where workers regularly discuss prevention issues may be precisely due to their greater concern about high levels of absenteeism. In fact, it is not new that worker participation is linked to worse health indicators. Previous studies have shown that organisations with collective representation of interests (indirect participation) sometimes have higher levels of occupational accidents because the absence of representation systems results in the non-reporting of certain occupational accidents [79,80,81]. In fact, in the European Working Conditions Survey (EWCS) of 2016, 46% of Spanish workers, and 42% for the European average, stated that they had worked while ill on some occasion in the last year [82]. The results of the EWCS and of the previous studies mentioned could explain the results obtained in the control variables of the present study, since both the visits of the labour inspection and the presence of workers’ representatives have been related to higher levels of absenteeism.

## 5. Conclusions

Currently, as a result of the changes that have taken place in the world of work, the level of exposure of workers to psychosocial risk factors has increased. From the point of view of occupational risk prevention, there is ample scientific evidence on the positive impact of the presence in workplaces of collective, indirect and representative representation through trade unions on the increase in preventive management rates and the reduction of occupational accidents. However, there is still no comparable scientific knowledge on the effect of direct worker participation on risks of psychosocial origin. In this context, the present study tries to contribute to this line of research by investigating the effect of direct participation of workers on the levels of absenteeism, based on the hypothesis that the simple fact of having documented the management of prevention does not guarantee the effectiveness of the prevention system, being necessary the inclusion of worker’s subjectivity in the management process to have positive effects on the level of absenteeism. In this sense, the statistical analyses carried out have shown how the passive participation of workers (information and training) in the preparation of prevention plans and on the evaluation of risks has a moderate impact on the levels of absenteeism, and it is necessary for direct participation to be active and at the end of the management process (participation in the design and implementation of preventive action measures on psychosocial risks) to guarantee a low level of absenteeism. In turn, the presence or agreement of preventive management indicators had no effect on absenteeism levels. However, in Spain, 88% of work centres have developed risk assessment because the law requires it, but only 34.5% of workers actively participate in its development, which shows that we can fall into an unrealistic institutional mirage of safety in which only bureaucratic compliance with the law is rewarded, and therefore active policies of coeducation or co-management are necessary to create a preventive culture within organizations that has a real positive impact on absenteeism. This study, is a first entry on the benefits of direct participation of workers that in future research can be developed through the study of the determinants that will influence the effectiveness of direct participation and equate its knowledge to that of indirect or representative participation.

## Figures and Tables

**Table 1:** Varibles Included in The Study.

Study Dimension	Questionnaire Questions
**Dependent variable**	Q450.- How would you describe the level of absenteeism at your workplace compared toother workplaces in the sector? Is it very high, quite high, within the average, quite low or very low?
**Independent variables**	
**Participation**	
*Passive participation*	Q256_5- Who has received the results of the workplace risk assessment? The workers themselves.
	Q163_3- On which of the following topics does your workplace provide training to workers? How to prevent psychosocial risks, such as stress or harassment
*Consultative participation*	Q358.- Are occupational risk prevention issues routinely discussed at meetings of personnel or equipment?
	Q350.- How often do representatives, workers and management talk about prevention of occupational risks?
*Active participation*	
	Q258b.- In case it is necessary to take action after a risk assessment. Are workers normally involved in its design and implementation?
	Q305.- Did workers participate in the design and adoption of measures to prevent psychosocial risks?
**Management**	
*Prevention plans*	Q156.- Is there a specific budget allocated each year to measures and equipment for the prevention of occupational hazards in your workplace?
	Q300.- Does your workplace have an action plan to prevent work-related stress?
	Q301.- Do you have a procedure for dealing with possible cases of harassment or bullying?
	Q302.- Do you have a procedure for dealing with possible cases of threats, insults or aggressions by clients, patients, students or other outsiders?
*Risks evaluation*	Q250.- Does your workplace routinely perform risk assessments in the workplace?
	Q252_5.- Which of the following aspects are normally included in these evaluations of risk in the workplace? Relationship between worker and supervisor
	Q252_6.- Which of the following aspects are normally included in these evaluations of risk in the workplace? Organizational aspects such as work schedules, breaks or work shifts
*Preventive planning*	Q303_1. In the last 3 years, has your workplace applied any of the following measures to prevent psychosocial risks? Reorganisation of work in order to reduce the demands and work pressure
	Q303_2.- In the last 3 years, has your workplace applied any of the following measures to prevent psychosocial risks? Confidential counselling for workers
	Q303_3.- In the last 3 years, has your workplace applied any of the following measures to prevent psychosocial risks? Implementation of a conflict resolution procedure
	Q303_4.- In the last 3 years, has your workplace applied any of the following measures to prevent psychosocial risks? Intervention in case of too many working hours or irregular schedule
**Control variables**	
*Collective representation*	Q166_1.- Which of the following forms of worker representation do you have in your workplace? Personnel delegate, works council or staff meeting
	Q166_2.- Which of the following forms of worker representation do you have in your workplace? Shop steward
	Q166_3.- Which of the following forms of worker representation do you have in your workplace? Prevention Delegate
	Q166_4.- Which of the following forms of worker representation do you have in your workplace? Occupational Safety and Health Committee
*Inspection Visits*	Q165.- Has your workplace received any visits from the Labour Inspectorate in the last 3 years to check compliance with risk prevention regulations? work?
*Economic sector*	[Sector]. sector of the workplace
*Workplace size*	[Size]. company size

Q450: question from ESENER-2 questionnaire number 450; Q256_5: question number 256_5; Q163_3: question number 163_3; Q358: question number 358; Q350: question number 350; Q258b: question number 258b; Q305: question number 305; Q156: question number 156; Q300: question number 300; Q301: question number 301; Q302: question number 302; Q250: question number 250; Q252_5: question number 252_5; Q252_6: question number 252_6; Q303_1: question number 303_1; Q303_2: question number 303_2; Q303_3: question number 303_3; Q303_4: question number 303_4; Q166_1: question number 166_1; Q166_2: question number 166_2; Q166_3: question number 166_3; Q166_4: question number 166_4; Q165: question number 165.

**Table 2:** Logistic regression multinomial for levels of absenteeism cited.

	Quite Low ^1^		Within Half ^1^		Very High Or Quite High ^1^	
	aOR(95%IC) ^2^	*p*-Value	aOR(95%IC) ^2^	*p*-Value	aOR(95%IC) ^2^	*p*-Value
**Participation**						
*Passive participation*						
Q256_5: Information						
Do not	1^3^		1^3^		1^3^	
Yes	**0.71 (0.57–0.87)**	**0.001**	**0.64 (0.51–0.79)**	**0.000**	0.97 (0.65–1.44)	0.869
Q166_3: Training						
Do not	1^3^		1^3^		1^3^	
Yes	**0.76 (0.61–0.94)**	**0.011**	0.88 (0.70–1.10)	0.258	1.04 (0.70–1.53)	0.863
*Consultative participation*						
Q358: Debates						
Do not	1^3^		1^3^		1^3^	
Yes	**1.24 (1.01–1.54)**	**0.049**	**1.71 (1.36–2.14)**	**0.000**	**1.54 (1.04–2.29)**	**0.033**
Q350: Speak						
Do not	1^3^		1^3^		1^3^	
Yes	0.91 (0.71–1.18)	0.483	0.82 (0.64–1.07)	0.145	0.82 (0.52–1.29)	0.396
*Active participation*						
Q258b: general risks						
Do not	1^3^		1^3^		1^3^	
Yes	0.87 (0.67–1.13)	0.295	**0.68 (0.52–0.89)**	**0.005**	0.90 (0.55–1.48)	0.690
Q305: psychosocial risks						
Do not	1^3^		1^3^		1^3^	
Yes	**0.66 (0.51–0.87)**	**0.003**	**0.76 (0.58–0.99)**	**0.049**	**0.43 (0.27–0.69)**	**0.000**
**Management**						
*Prevention plans*						
Q156: budget						
Do not	1^3^		1^3^		1^3^	
Yes	1.13 (0.93–1.38)	0.225	1.12 (0.91–1.38)	0.295	0.99 (0.68–1.43)	0.936
Q300: Stress						
Do not	1^3^		1^3^		1^3^	
Yes	0.87 (0.58–1.30)	0.505	1.04 (0.69–1.56)	0.862	0.70 (0.35–1.41)	0.314
Q301: Harassment						
Do not	1^3^		1^3^		1^3^	
Yes	**1.69 (1.14–2.49)**	**0.008**	1.05 (0.69–1.60)	0.823	1.03 (0.52–2.06)	0.926
Q302: Threats						
Do not	1^3^		1^3^		1^3^	
Yes	1.24 (0.79–1.92)	0.348	1.37 (0.86–2.19)	0.191	1.44 (0.68–3.06)	0.339
*Risks evaluation*						
Q250: Overall assessment						
Do not	1^3^		1^3^		1^3^	
Yes	1.42 (0.96–2.11)	0.082	1.49 (0.99–2.24)	0.058	0.65 (0.32–1.34)	0.246
Q252_5: Relationships						
Do not	1^3^		1^3^		1^3^	
Yes	1.24 (0.98–1.58)	0.076	1.29 (1.00–1.65)	0.054	1.09 (0.70–1.70)	0.695
Q252_6 Organization						
Do not	1^3^		1^3^		1^3^	
Yes	0.97 (0.76–1.23)	0.795	0.95 (0.74–1.22)	0.665	1.29 (0.82–2.05)	0.271
*Preventive planning*						
Q303_1: work pressure						
Do not	1^3^		1^3^		1^3^	
Yes	1.09 (0.86–1.38)	0.344	0.95 (0.74–1.22)	0.695	1.34 (0.88–2.06)	0.173
Q303_2: Consulting						
Do not	1^3^		1^3^		1^3^	
Yes	**1.41 (1.10–1.81)**	**0.007**	**1.30 (1.01–1.68)**	**0.050**	1.17 (0.75–1.85)	0.488
Q303_3: Conflicts						
Do not	1^3^		1^3^		1^3^	
Yes	1.06 (0.81–1.39)	0.651	**1.40 (1.07–1.83)**	**0.015**	1.31 (0.81–2.10)	0.231
Q303_4: Schedules						
Do not	1^3^		1^3^		1^3^	
Yes	0.96 (0.72–1.26)	0.746	0.96 (0.72–1.27)	0.757	1.06 (0.65–1.72)	0.819
**Control variables**						
*Collective representation*						
Q166_1: Personal Delegate						
Do not	1^3^		1^3^		1^3^	
Yes	1.13 (0.86–1.47)	0.384	**1.34 (1.02–1.77)**	**0.036**	**2.01 (1.27–3.19)**	**0.003**
Q166_2: Delegate union						
Do not	1^3^		1^3^		1^3^	
Yes	0.94 (0.71–1.25)	0.661	1.24 (0.93–1.65)	0.136	0.75 (0.46–1.22)	0.246
Q166_3: D. Prevention						
Do not	1^3^		1^3^		1^3^	
Yes	0.97 (0.77–1.21)	0.791	0.94 (0.75–1.19)	0.605	0.83 (0.54–1.26)	0.376
Q166_4: Safety Committee						
Do not	1^3^		1^3^		1^3^	
Yes	1.08 (0.81–1.44)	0.609	1.24 (0.93–1.66)	0.142	1.57 (0.97–2.54)	0.067
*Q165: Inspection Visits*						
Do not	1^3^		1^3^		1^3^	
Yes	1.01 (0.83–1.23)	0.900	**1.91 (1.56–2.33)**	**0.000**	**2.02 (1.41–2.90)**	**0.000**
*Economic sector*						
Health	1^3^		1^3^		1^3^	
farming	1.08 (0.66–1.77)	0.754	1.21 (0.72–2.05)	0.469	1.06 (0.36–3.10)	0.913
Building	1.43 (0.94–2.17)	0.092	1.27 (0.81–2.00)	0.298	2.03 (0.92–4.47)	0.078
Industry	1.26 (0.85–1.87)	0.256	1.35 (0.88–2.07)	0.165	1.55 (0.71–3.37)	0.270
Commerce	0.90 (0.65–1.25)	0.540	1.14 (0.80–1.62)	0.477	1.36 (0.70–2.62)	0.361
Administration	1.03 (0.73–1.45)	0.889	1.06 (0.72–1.55)	0.787	1.73 (0.87–3.43)	0.118
Public	1.34 (0.77–2.33)	0.296	1.42 (0.79–2.56)	0.237	1.72 (0.63–4.74)	0.292
*Workplace size*						
250 or more	1^3^		1^3^		1^3^	
50–249	0.64 (0.18–2.30)	0.498	0.57 (0.17–1.88)	0.355	0.43 (0.09–2.04)	0.289
10–49	0.39 (0.11–1.36)	0.138	**0.22 (0.07–0.71)**	**0.012**	**0.19 (0.04–0.89)**	**0.035**
09.06	**0.25 (0.07–0.88)**	**0.031**	**0.34 (0.07–0.79)**	**0.019**	**0.15 (0.03–0.71)**	**0.017**
Chi squared	389.705	0.000				
Cox and Snell R2	0.119					
R2 Nagelkerke	0.133					
Population	3.162					
Number of valid cases and percentage	2916 (92.2%)					

^1^ The reference category is the very low level of absenteeism; ^2^ aOR: Odds ratios adjusted for other variables of participation, management and contral, 95% CI: confidence interval of 95%.; ^3^ Reference category for each variable.; Numbers that are statistically significant have been highlighted in bold; Q256_5: question from ESENER-2 questionnaire number 256_6; Q163_3: question number 163_3; Q358: question number 358; Q350: question number 350; Q258b: question number 258b; Q305: question number 305; Q156: question number 156; Q300: question number 300; Q301: question number 301; Q302: question number 302; Q250: question number 250; Q252_5: question number 252_5; Q252_6: question number 252_6; Q303_1: question number 303_1; Q303_2: question number 303_2; Q303_3: question number 303_3; Q303_4: question number 303_4; Q166_1: question number 166_1; Q166_2: question number 166_2; Q166_3: question number 166_3; Q166_4: question number 166_4; Q165: question number 165.

## Appendix A

**Table A1:** Descriptive analysis of the multinomial logistic regression.

	Number (%)	Test of the Likelihood Ratio	
		Chi Squared	*p*-Value
**Level of absenteeism**			
very low	1663 (54.0)		
quite low	650 (21.1)		
Within half	614 (19.9)		
Very high or quite high	151 (4.9)		
**Participation**			
*Passive participation*			
Q256_5: Information		21.970	0. 000
Do not	1417 (46.0)		
Yes	1661 (54.4)		
Q166_3: Training		7.080	0. 069
Do not	1605 (52.2)		
Yes	1473 (47.8)		
*consultative participation*			
Q358: Debates		23.963	0. 000
Do not	1631 (53.0)		
Yes	1447 47.0)		
Q350: Speak		2.492	477
Do not	2215 (72.0)		
Yes	863 (28.0)		
*Active participation*			
Q258b: general risks		7.916	0. 048
Do not	863 (28.0)		
Yes	2215 (72.0)		
Q305: psychosocial risks		18.878	0. 000
Do not	2023 (65.7)		
Yes	1054 (34.3)		
**Management**			
*Prevention plans*			
Q156: budget		2.152	542
Do not	1256 (40.8)		
Yes	1821 (59.2)		
Q300: Stress		1.680	0. 641
Do not	272 (8.9)		
Yes	2805 (91.1)		
Q301: Harassment		7.893	0. 048
Do not	2730 (88.7)		
Yes	348 (11.3)		
Q302: Threats		2.175	0. 537
Do not	2873 (93.4)		
Yes	205 (6.6)		
*Risks evaluation*			
Q250: Overall assessment		7.986	0. 046
Do not	2735 (88.9)		
Yes	343 (11.1)		
Q252_5: Relationships		5.403	145
Do not	1595 (51.8)		
Yes	1483 (48.2)		
Q252_6 Organization		1.719	633
Do not	1441 (46.8)		
Yes	1636 (53.2)		
*preventive planning*			
Q303_1: work pressure		2.774	428
Do not	1846 (60.0)		
Yes	1232 (40.0)		
Q303_2: Consulting		8.651	0.034
Do not	2119 (68.8)		
Yes	959 (31.2)		
Q303_3: Conflicts		6.489	0.090
Do not	2242 (72.8)		
Yes	836 (27.2)		
Q303_4: Schedules		261	967
Do not	2515 (81.7)		
Yes	563 (18.3)		
**Control variables**			
*collective representation*			
Q166_1: Personal Delegate		11.108	0. 0011
Do not	2030 (66.0)		
Yes	1048 (34.0)		
Q166_2: Delegate union		5.103	0. 164
Do not	2299 (74.7)		
Yes	779 (25.3)		
Q166_3: D. Prevention		932	818
Do not	1542 (50.1)		
Yes	1535 (49.9)		
Q166_4: Safety Committee		4.551	208
Do not	2491 (80.9)		
Yes	586 (19.1)		
*Q165: Inspection Visits*		58.820	0. 000
Do not	1700 (55.2)		
Yes	1378 (44.8)		
*Economic sector*		16.416	0. 564
Health	345 (11.2)		
farming	167 (5.4)		
Building	285 (9.3)		
Industry	344 (11.2)		
Commerce	1188 (38.6)		
Administration	638 (20.7)		
Public	111 (3.6)		
*Size workplace*		48.745	0. 000
250 or more	29 (0.9)		
50–249	220 (7.1)		
10–49	1195 (38.8)		
09.06	1635 (53.1)		

Q256_5: question from ESENER-2 questionnaire number 256_6; Q163_3: question number 163_3; Q358: question number 358; Q350: question number 350; Q258b: question number 258b; Q305: question number 305; Q156: question number 156; Q300: question number 300; Q301: question number 301; Q302: question number 302; Q250: question number 250; Q252_5: question number 252_5; Q252_6: question number 252_6; Q303_1: question number 303_1; Q303_2: question number 303_2; Q303_3: question number 303_3; Q303_4: question number 303_4; Q166_1: question number 166_1; Q166_2: question number 166_2; Q166_3: question number 166_3; Q166_4: question number 166_4; Q165: question number 165.
